# Simulation-Based Rule Generation Considering Readability

**DOI:** 10.1155/2015/159289

**Published:** 2015-03-24

**Authors:** H. Yahagi, S. Shimizu, T. Ogata, T. Hara, J. Ota

**Affiliations:** ^1^Research into Artifacts, Center for Engineering (RACE), The University of Tokyo, 5-1-5 Kashiwanoha, Kashiwa, Chiba 277-8568, Japan; ^2^ANA Strategic Research Institute, 1-5-2 Higashi-Shinbashi, Minato-ku, Tokyo 107-7140, Japan

## Abstract

Rule generation method is proposed for an aircraft control problem in an airport. Designing appropriate rules for motion coordination of taxiing aircraft in the airport is important, which is conducted by ground control. However, previous studies did not consider readability of rules, which is important because it should be operated and maintained by humans. Therefore, in this study, using the indicator of readability, we propose a method of rule generation based on parallel algorithm discovery and orchestration (PADO). By applying our proposed method to the aircraft control problem, the proposed algorithm can generate more readable and more robust rules and is found to be superior to previous methods.

## 1. Background

### 1.1. Aircraft Control at an Airport

Due to the effects of rapid globalization in recent years, large airports around the world, including Narita Airport in Japan, are forced to increase the number of takeoffs and landings they handle. Therefore, taxiing aircraft in the airport, which causes congestion, has been an issue of concern. Many have sought to improve the operation method for dealing with this crowding.

The taxiing of aircraft is controlled by ground control (GC), which manages taxiing near the terminal, and air traffic controllers, who look down upon the entire airport from the control tower.

**Figure 1:**
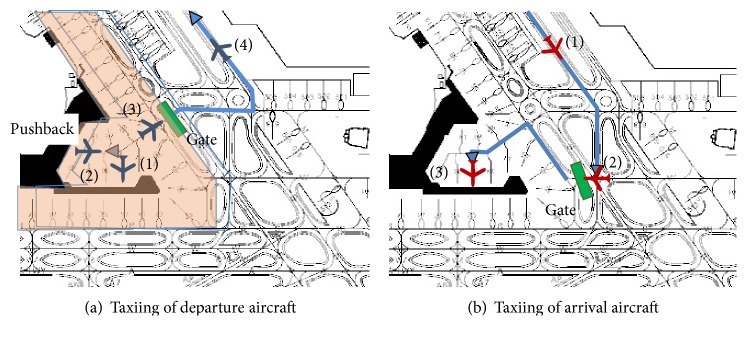
Taxiing aircraft.

To start, as shown in Figure 1(a), departing aircraft “pushback” on instruction from GC from the point called “spot” to passenger embarkation and debarkation as shown from (1) and (2). Pushback is an operation that pulls an aircraft that structurally cannot taxi backward. In response to instructions for the route to the runway from the air traffic controllers after the pushback, from gate, which as the entrance of the ramp area, aircraft move from (3) out of the ramp area near the terminal, travel along it to the runway as shown in (4), and take off.

Arriving aircraft, as shown in Figure 1(b), land on the runway in (1), receive instructions for taxiing route from air traffic controllers, and travel to the gate shown in (2). Then, GC issues a permit of entry for the ramp area at the right time for any state aircraft or vehicle that may be driving through the ramp area to the spot of interest shown in (3).

GC must control the aircraft (of which there are more than 30 at the same time in an airport at peak times) quickly and safely to allow these aircraft to taxi. For example, as the highlighted areas of Figure 1(a) have a bidirectional travel route, there is a need for appropriate control to ensure that deadlock does not occur. For ease of traffic congestion, it is necessary to direct all in concert to control the start timing of the pushback, and the entry timing of the ramp area. The task is performed heuristically, but there are currently concerns as to if the heuristics can cope with a future increase in flights. Therefore, it is required to computationally generate control rules of GC, for variety in schedules of aircraft, which we call robustness of the solution in this paper.

### 1.2. Related Work

The aircraft control problem can be regarded as an optimization problem of constructing decision-making rules for a multiagent system [1, 2]. This term refers to a system that hopes to achieve complex goals through the operation of multiple agents repeating actions based on a set of rules and observation of the environment. There are a variety of examples present in the literature other than aircraft control.

In [3, 4], they introduced the concept of using agent-based systems for the construction of a flexible production system with high efficiency. They define two kinds of agents: the first one is to make a work plan for each device and the second one is to integrate the plans. In the paper, they design behavioral rules for the agents and measure the efficiency of the system as a whole.

In [5], multiagent system is proposed for traffic flow maximization via traffic signal control. With each traffic signal independently determining the congestion state of its surroundings, it can continuously change the time interval of the signal accordingly and can be successfully efficient at maximizing traffic flow as a result.

In [6], they model an agent-based system for workflow involving business loans from a bank, such as requests received from customers. This system independently models the performance of actions such as requests received by each agent.

Decision-making rules in any agent-based systems such as these, where decisions are not reversible in all situations, must not just be robust but in light of maintenance must also be very readable.

The proposed system here for controlling aircraft is intended to be implemented by humans using rules. As a result, it is necessary to calculate highly readable rules that can be operated and understood by humans.

For the above reasons, it is important to use a method of generating behavioral rules for multiagent system that takes into account both robustness and readability.

From the viewpoint of the robust optimization problem, in [7], a robust optimization technique is proposed for known linear programming problems with variation. Deterministic algorithms guarantee the robustness of calculating solutions, but a modeled problem must be linear to be solved. For many systems, application to nonlinear problems is difficult to realize, and robust optimization techniques using simulation are required. In [8], a robust optimization technique with Monte Carlo method is presented. In this method, simulations are repeatedly performed by using random numbers, and the robustness of the solution is calculated from the results. However, if the simulation is made on a large scale, computation time becomes enormous, and the accuracy of any solution calculated within a realistic time is very low. In [9, 10] they propose methods of reducing the number of simulations by combining it with a curved surface approximation of the evaluation function through response surface methodology. However, these methods have been applied to problems with continuous solution space, and applying them to discrete multimodal problems such as rule generation is difficult. Further, as described above, there is still no method that contains measures to reduce the number of simulations without surface approximation, which would be necessary for rule generation for multiagent systems.

In [11], an approximate optimization method is utilized on problems with a tree-structure solution that mimics the evolution of organisms called genetic programming (GP). This optimization technique has been studied many times, its rules taking the form of polynomial conditions. However, this method suffers from a phenomenon called bloat, where solutions become increasingly complicated; it calculates solutions with low readability. In order to suppress bloat, in [12], a method is proposed for setting a new evaluation function that combines the depth of the tree structure with what the solution evaluation function should be. Further, in [13], a method is presented of solving a multiobjective optimization problem treating the depth of the tree structure as a new evaluation function. However, these methods are discussed only in terms of improving the robustness of the rule, with no consideration towards the readability of the solution. In [14], rules are generated by optimizing tempo and pitch to match user preferences, which is represented as a polynomial using arithmetic operations, and so forth, over several parameters, but it has very low readability.

From the point of view of software maintenance, in [15], they enumerate about twenty different elements that affect the readability of source code of a computer program, and in [16], an evaluation expression for measuring element readability is proposed. However, few studies have applied these metrics to rule generation.

In addition, in [17], although a rule generation method for multiagent systems has been proposed, evaluation was not sufficient from the viewpoint of the scale of the problems to evaluate.

### 1.3. Purpose of This Study

As shown in the previous section, research on rule generation has been focused on optimization from the viewpoint of a kind of efficiency or robustness, with little focus on readability. Therefore, the purpose of this study is the construction of a simulation-based rule generation method that considers both robustness and readability.

In this paper, we propose a method that incorporates a method both with PADO [18] and with Operator Equalization [19] using metrics on readability. Then, we apply our method to the aircraft control problem in two settings and evaluate the results.

## 2. Target Problem and Readability

### 2.1. Problem Settings

We use the following situation as the problem setting of this study. There is more than one agent in the environment, with each agent performing observations to produce the state space based on the information obtained. An action is decided using predetermined rules based on the state space, and this is repeated several times. We assume that the state is defined in advance in accordance with the problem, that any error in the environment that may occur is known, but that there is no error when retrieving information. What actions the agents may select is defined in advance according to the type of problem and on the basis of rules. We deal with the problem of obtaining the appropriate rules for this environment. We evaluate the rules in the simulation environment multiple times with different settings. In this paper, as an evaluation value, we utilize total stopping time of all the agents (aircraft), which does not take negative value and whose optimal number is equal to zero (so-called s-type characteristics). Then we adopt the index for measuring the robustness of the rules, as shown in

(1) $$\begin{array}{c} \text{Robustness}=\log\left(\frac{1}{\sum E^{2}}\right),\\ E\text{:}\;\text{Evaluation value in each simulation.} \end{array}$$

In addition, our rules are evaluated using the readability formula described in Section 2.2.

### 2.2. Readability Formula

In this study, we evaluate the rules using the readability formula proposed by [16]

(2) Readability=11+e−z,z=8.87−0.033V+0.40  Lines−1.5  Entropy,V:  Halstead's Volume  =ProgramLength∗log⁡⁡ProgramVocabulary,ProgramLength:  Number  of  words  in  the  documents,ProgramVocabulary:  Number  of  different  words  in  the  documents,Lines:  Number  of  lines,Entropy:  HX=−∑i=1npxilog⁡2pxi,pxi=count(xi)∑j=1ncount(xj),countx:  Number  of  appearances  of  the  word  “x”,n:  Number  of  words.

This formula measures the readability of source code through a variety of indicators. In formula (2), *V* is an index called Halstead's Volume [20] that represents the complexity of the document as a function of the length of the document, increasing with complexity. Line represents the number of lines in the document, which we have quantified as the number of rows. Entropy represents the amount of information in the document, which takes an increasing value as the probability of occurrence of each word falls.

## 3. Proposed Method

### 3.1. Rule Structures and Readability

According to the readability index mentioned in the previous section, line breaks in the document help to improve readability. Therefore, we propose a hierarchical two-step representation of rules, condition and action. Here we consider that the hierarchy corresponds to the line breaks. We then represent the rules by a tree-structure inequation or equation, to graphically represent the order of evaluation.

**Figure 2:**
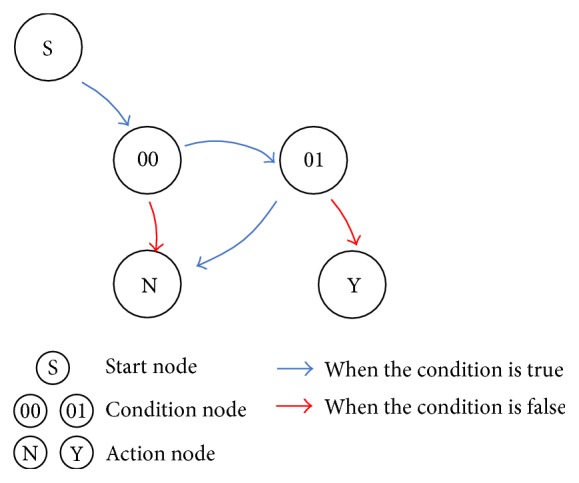
A rule expressed by a graph.

The graph determines whether the agent starts to take a specific action or not, such as to start moving. The graph has three types of nodes: a start node, condition nodes, and action nodes, as shown in Figure 2. Condition nodes are associated with equality/inequality conditions, which are represented by trees as later defined, and action nodes are associated with whether the agent to take the action (Y) or not (N). A condition node has an outdegree of two, represented by the arrows. On receipt of a state vector, the rule outputs whether to take a single action or not. The state vector is given as input, starting from the start node and arriving at the condition node. The process then proceeds to the next node, following the red arc if false or the blue arc if true. This is repeatedly performed until arriving at an action node. The process outputs the behavior associated with the action node when it arrives at the node, and that action is then taken by the previously defined agent.

**Figure 3:**
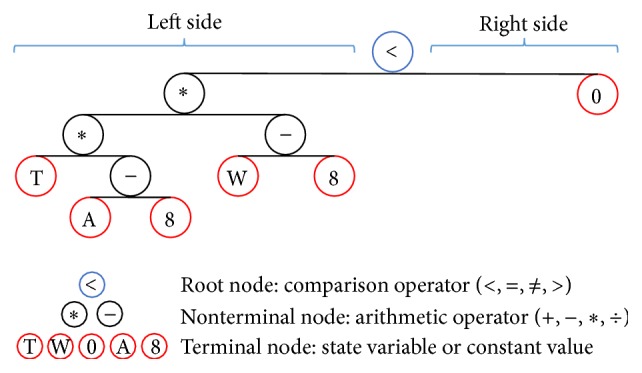
A condition function expressed by a tree.

The equality/inequality conditions associated with the condition nodes of the graph are represented by trees consisting of three kinds of nodes, root nodes, nonterminal nodes, and terminal nodes, as shown in Figure 3. The root node represents a comparison operator (<, =, ≠, and >), the right-hand side represents a constant (in this tree, 0), and the left-hand side represents arithmetic operators, a decomposition of a polynomial in the state variables and constants. Terminal nodes on the left-hand side represent the state variables or constants, while nonterminal nodes represent arithmetic operators. When evaluating a formula represented in such a tree, the state vector is input, and the result calculated by substituting the values of the state variables in the expression and using the comparison operator of the root node against 0 on the right side is output. In the case of Figure 3, it expresses an inequality equation of *T*∗(*A* − 8)∗(*W* − 8) < 0, where *A*, *T*, and *W* are state variables.

Table 1 describes the variables appearing in formula (2) and their correspondence to both the source code and the representation of rules. Here we understand that the rules are similar to the source codes in that humans need to read and understand them.

In this representation, since the total combination of tree and graph forms a ruleset corresponding to the entire document in the source code, we associate the nodes in the graph with lines of source code and treat the number of nodes across all conditional trees as the number of words.

### 3.2. Details of Proposed Method

**Figure 4:**
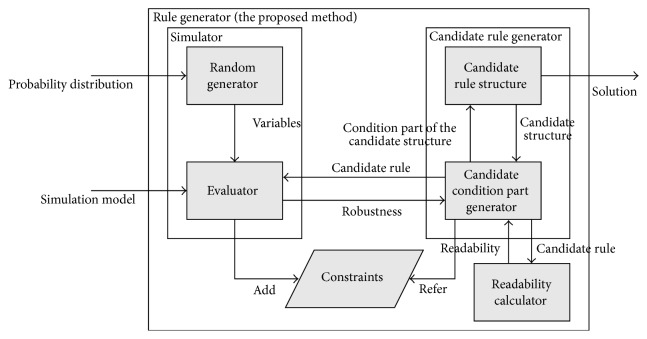
Description of the proposed method.

We base our method on the method proposed in [17]. An overall picture of the algorithm is shown in Figure 4.

By the problem setting described in the previous section, the simulation model to be calculated is our input, and the algorithm calculates a set of rules as output. It is assumed that the simulation model at this time meets the requirements as described in the previous section.

The main components of the algorithm are the following four sections.

- Simulator;
- Candidate Rule Generator;
- Constraints;
- Readability Calculator.

In the Simulator, using the simulation model as an input, our method generates an evaluation solution using candidate rules also serving as input and outputs the evaluation value using an index that is defined in advance. The Readability Calculator calculates the readability of each candidate solution. As we repeatedly create new solution candidates, we approach the optimal solution. Without attachment to generate a solution structure that is layered as described in the previous section, the solution candidate generator will be in a hierarchical structure. Further, in the constraint section, every time the simulation is performed, we automatically extract and save the constraints of the rules for a feasible solution. Then, in generating a new solution candidate by regenerating and referencing previous solution candidates, they must also satisfy these constraints. This part is utilized for avoiding deadlocks and inefficient behavior of the agent.

The flow of the algorithm is outlined below.

When calculation first begins, the graph portion of the solution candidates is generated by the graph generating unit. Then, it generates a set of trees (equal to the number of conditional nodes) in the amount required for the graph tree generator. For each candidate solution, we evaluate the simulator, with random variables varying according to the distribution of each. The simulation is performed multiple times, and the robustness of the candidate solution calculated according to formula (1).

We use a local search method to generate trees. We believe that an infeasible solution that cannot be calculated becomes feasible with repeated minor modifications of the tree through rule generation, and the evaluation value falls largely within the solution space.

After the evaluation value of some graphs is calculated in this manner, we use the gathered information to produce an improved graph using a technique known as PADO, first proposed by [18], and through a method called Operator Equalization. This method is described in the next section.

Finally, we can calculate the optimal solution by repeating the above processes.

### 3.3. Operator Equalization

When improving robustness and readability is treated as a multiobjective optimization problem, such as in [17], the problem, as stated in [21], will fall into local optimal solutions, which undesirably lowers the quality of the solution. In this study, we prevent this by building in optimization of the graph by PADO via a technique called Operator Equalization. This technique, first proposed in [19], has been suggested as a method of suppressing the excess complication of solutions through GP via the most fundamental tree optimization methods. With this method we take into account both the robustness and the complexity of each individual in the previous generation in generating the children that make up the next generation of rules. Like GP, PADO as used in this method is also a method that approximates the solution by mimicking natural selection; in this case, it is akin to repeated cross survival selection and mutation by performing optimization of the graph by the concept of Operator Equalization. We show the algorithm in a concrete fashion below.

**Figure 5:**
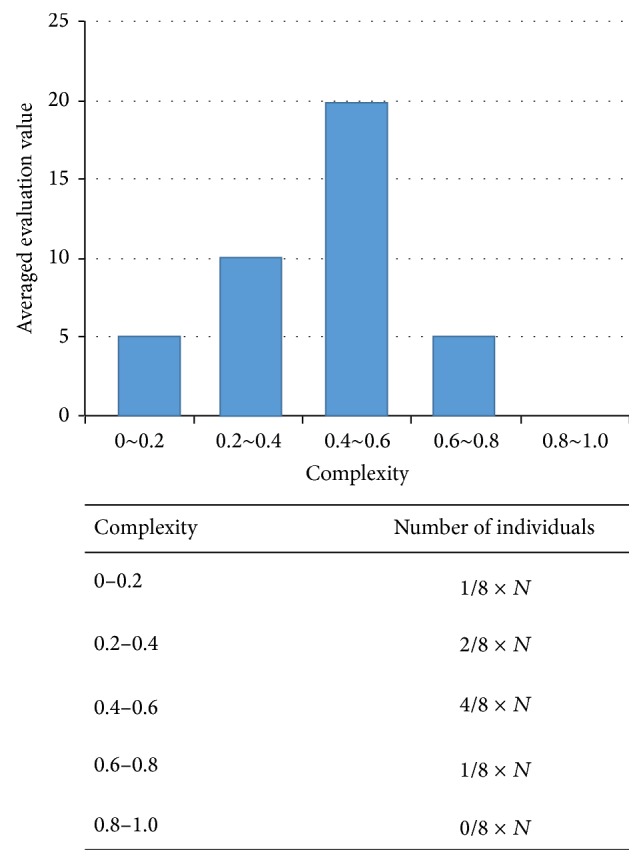
Distribution of next generation.

Creating a distribution of evaluation values from readability was calculated by applying formula (2) to the current generation, such as shown in the upper graph in Figure 5. To determine the distribution of the complexity of the next generation individual using this distribution, the next generation is allocated in proportion to the magnitude of evaluation values for the complexity of the previous generation. That is, assuming total population in PADO as *N*, it is determined in the ratio, as shown in the lower table in Figure 5.

**Figure 6:**
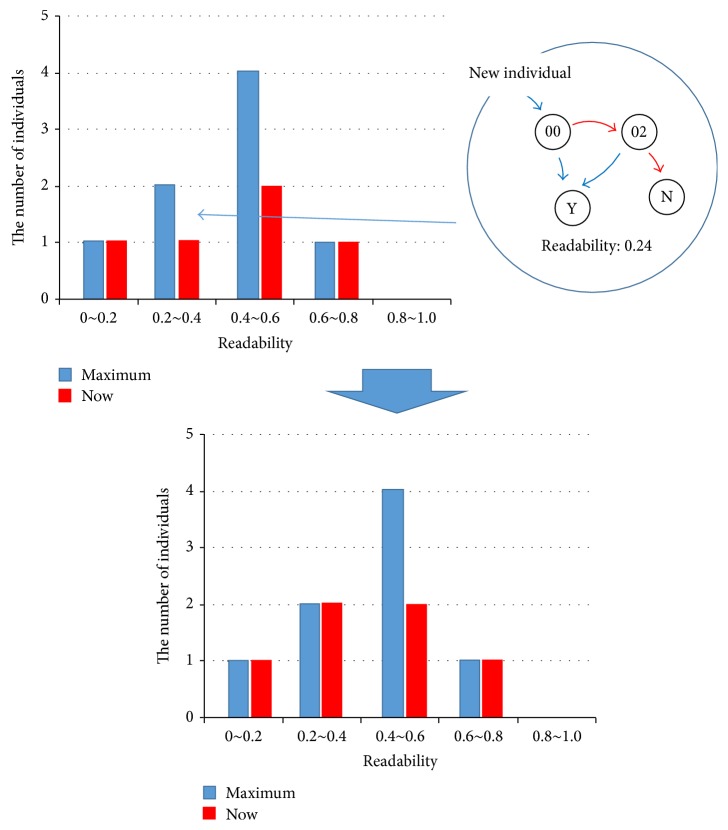
Saving of new individuals.

After the distribution has been determined, individuals are produced by crossing the individual child candidates, to perform the calculation of the evaluation value of a decision tree that will lead to a condition node by the local search method described above and to calculate the readability by the readability index. For example, in case of Figure 6, *N* is set as 7 and maximal numbers of each category are obtained in advance, as 1 for the category whose readability band is between 0 and 0.2, 2 for the band between 0.2 and 0.4, 4 for the band between 0.4 and 0.6, 1 for the band between 0.6 and 0.8. Then new individual is obtained as a 6th candidate, whose readability is 0.24. The individual can be assigned to the band between 0.2 and 0.4 because it does not exceed the number of individuals allocated to the band.

The graphs are first selected not to exceed the number of individuals allocated to the corresponding readability band in Figure 5. If the value of formula (1) for a certain individual is best among others and it violates the readability band when it is selected, the graph is selected by slightly changing the number of individuals allocated to each band.

## 4. Evaluation

### 4.1. Problem Setting for Evaluation

Simulations are performed using the entire Narita Airport to evaluate the performance of the proposed method, by showing how the proposed one can generate feasible control rules of taxiing aircraft. The evaluation is made by comparison with the present control rule in Narita Airport and that with other methods.

**Figure 7:**
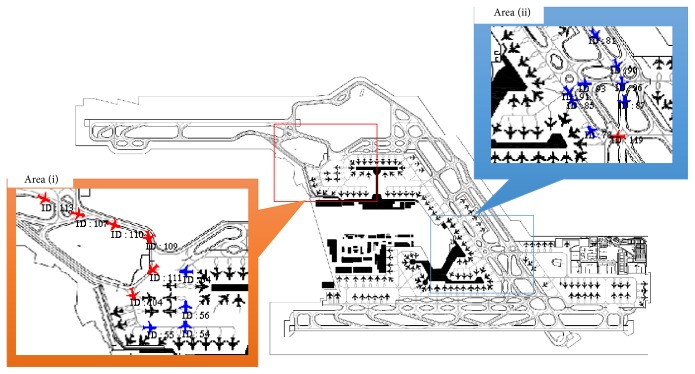
Two congested areas in the simulated environments.

Figure 7 represents the entire Narita Airport, with about 300 spots. As a result of discussion with the airport officials, it was found that there are two specific local areas that most required an improved control method for congestion relief, as shown in Figure 7.

In Area (i) of Figure 7, aircraft begin with a pushback as they attempt to start out. Attempts to head to or from the runway are complicated, and nearly deadlock situation of the loop due to pushback operations can force the expensive act of waiting increasingly long in the air while the runway is unusable due to a waiting column of aircraft. Therefore, the aim is to alleviate congestion by controlling the proper timing of the pushback in this spot.

In Area (ii) of Figure 7, as described in Section 1, the ramp area above the gate is a bidirectional traveling path. Aircraft must wait at this gate point depending on the status of the ramp area, and so forth, for deadlock prevention. However, there is a possibility that waiting on the spot so long will stop the travel of the aircraft, which leads to congestion. Therefore, the aim is to alleviate congestion by optimizing the wait-passing rules of the gate.

The control rules of these two areas affect each other, and so it is necessary to design them simultaneously with each other. Evaluating the proposed method by using a multiagent system model as described above will be with reference to the following: the specific scheme in control of gate control and pushback and the state variables used in each rule.

*(i) Control of Pushback*. Departing aircraft at the spots asks for instruction to the air traffic controllers when it becomes their departure time. Air traffic controllers issue instructions to either start or wait in order starting with the aircraft that have been waiting longest. The aircraft receives an instruction either to start the pushback or to report back to the controller after a certain time. This time interval is equal to the minimum time interval in the simulation, which is set to one second in the present study. 

*〈State Variables〉*. Consider the following.

① The number of taxiing aircraft in Area (i): expressed as  AirN.
② The number of arrivals coming to Area (i): expressed as  UseSp.
③ The number of aircraft pushing back in Area (i): expressed as  PushN.
④ The number of waiting aircraft in Area (i): expressed as  WaitN.
⑤ The waiting time of the departing aircraft: expressed as  Time.

*(ii) Control of the Gate*. When the aircraft passes through the appropriate gate, it reports to the air traffic controllers. The air traffic controllers evaluate the rules and instruct the aircraft to either pass or wait. An aircraft that receives the instruction to pass enters the ramp area to pass through the gate. An aircraft that receives the instruction to wait must wait to report back to the controller after a certain time. This time interval is equal to the minimum time interval in the simulation, which is set to one second in the present study.

*〈State Variables〉*. Consider the following.

① Whether the aircraft is waiting on each spot: expressed as  Si_Sn  (1 when waiting at spot *i*, 0 otherwise).
② Whether the aircraft is pushing back on each spot: expressed as  Si_Pb  (1 when with pushback at spot *i*, 0 otherwise).
③ The waiting time of an aircraft on each spot: expressed as  Si_St  (waiting time at spot *i*).
④ The waiting time of an aircraft on the stop line: expressed as  Sus_St.
⑤ The number of waiting aircraft by the stop line: expressed as  Sus_Sn.
⑥ Whether an aircraft is taxiing on the ramp area: expressed as  OnRamp (1 when taxiing on the ramp area, 0 otherwise).
⑦ The kind of aircraft (departing or arriving) taxiing on the ramp area: expressed as  RampType  (1 when the departing aircraft is taxiing, 2 when arriving aircraft is taxiing, 0 otherwise).

We assume this simulation is during the busy time in Narita, time slot from 13:30 to 19:30, when the departing aircraft and arriving aircraft are equally three per 30 minutes, which means that totally six aircraft are departing or arriving within 30 minutes in the airport. We run the simulations a fixed number of times (50 in this paper), with each candidate rule system, setting *N* (total population in PADO) = 20, using the characteristic of total stopping time of all aircraft as the evaluation value for the robustness of the set of rules as a solution candidate as in formula (1). Departing and arriving times of all aircraft determined randomly assuming uniform distribution in 30 minutes.

### 4.2. Result

**Figure 8:**
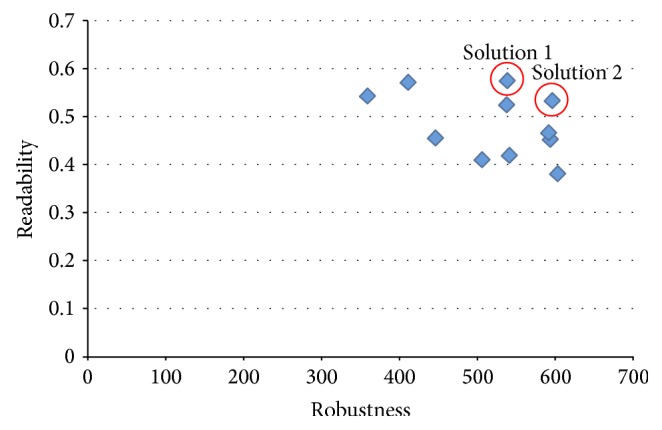
Solutions on the last generation.

The result of the above is shown in Figure 8, showing that it was possible to calculate the Pareto optimal solution of the two problems described above.

**Figure 9:**
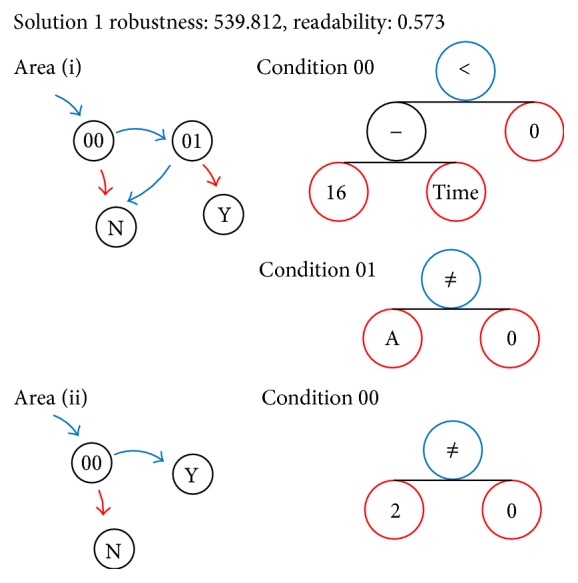
Graph expression of Solution 1.

*〈Solution 1〉 (Figure 9)*. The rule at the start of the pushback region in the solution of (i) is to wait if it has not been over 16 seconds, and if it has been 16 seconds or more, then proceed to evaluation of condition 01. The aircraft can start only after waiting even if empty, and then only if no aircraft is taxiing (represented by the variable *A* corresponding to state variable 1).

In the solution of (ii), since the condition node 00 is always true in any state, the rule for passing through to the gate region in Area (ii) is to always allow entry.

This solution was the most readable among solutions, and when the simulation was performed using these rules, the probability that deadlock did not occur was 99.1%.

**Figure 10:**
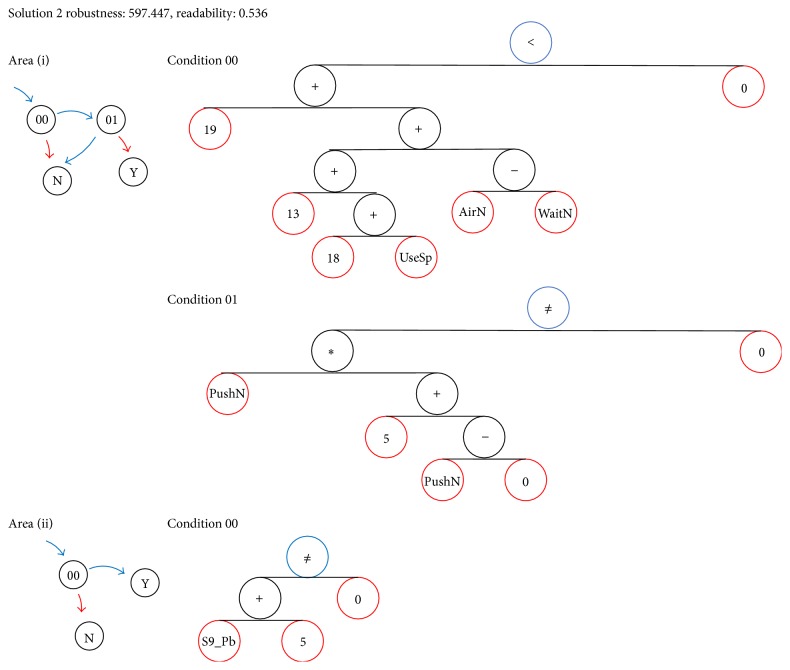
Graph expression of Solution 2.

*〈Solution 2〉 (Figure 10)*. The rule at the start of the pushback region in the solution of (i) involves the variables  AirN  (the amount of aircraft taxiing),  UseSp  (the number of arrivals coming to Area (i)), and  WaitN  (the number of waiting aircraft). This condition can be interpreted as a rule that depends solely on the relationship between  WaitN  and  AirN  regardless of  UseSp;  if  AirN  is greater than or equal to  WaitN, conditional expression 00 is false, and if  AirN  is less than  WaitN, conditional expression 00 is true. The agent instructs the aircraft to wait if the condition is found to be false but proceeds to evaluation of condition 01 if true. Condition 01 is a second inequality containing an additional state variable  PushN  (the number of aircraft pushing back in Area (i)) but, given that  PushN  is over the natural numbers, this condition ends up equivalent to the rule that  PushN  is greater than 0. Conditional expression 01 is then to wait if there are no aircraft pushing back and start otherwise.

The solution for Area (ii) changes according to the value of the variable  S9_Pb  (1 when with pushback at spot 9, 0 otherwise). Because the range of this variable is 0-1, condition 00 is always true. Thus this is effectively equivalent to always pausing in the same manner as the previous solution; it is a rule that passes through the line.

This solution has the second best scores in both robustness and readability among the calculated Pareto solutions. The probability that these rules do not generate the deadlock was determined by simulation to be 97.9%.

Such rules would have been hard to produce by hand but could be obtained by using the proposed method. As for the current control rules of Narita Airport, an aircraft is allowed to start when it reaches Area (i), and it is passed through the gate if there are no aircraft ramps in Area (ii). However, under this rule, according to the results of our simulation, the probability that deadlock does not occur is a mere 60%. The number of takeoffs and landings at Narita Airport continues to grow every year, and it is expected to increase by a factor of 1.5 from 2012 to 2015. This rule was created in 2012, when it was not possible to assume an increase in the departure and arrival numbers and has more recently had a low executable probability. Enormous cost could result under the present method, but with the results of the proposed method, the chance of avoiding deadlock is about 99%, which shows the effectiveness of the proposed method.

### 4.3. Comparison with the Former Method

The experiments were carried out by comparing the proposed method and the one proposed in [17]. We perform each approach over the course of 10 hours and compare the robustness and readability of the final solutions.

**Figure 11:**
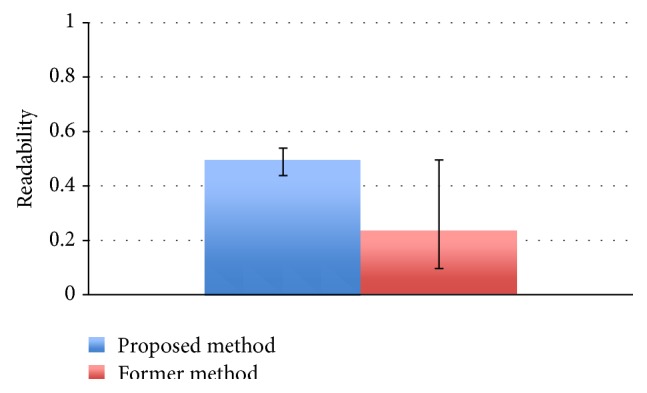
Comparison of readability.

**Figure 12:**
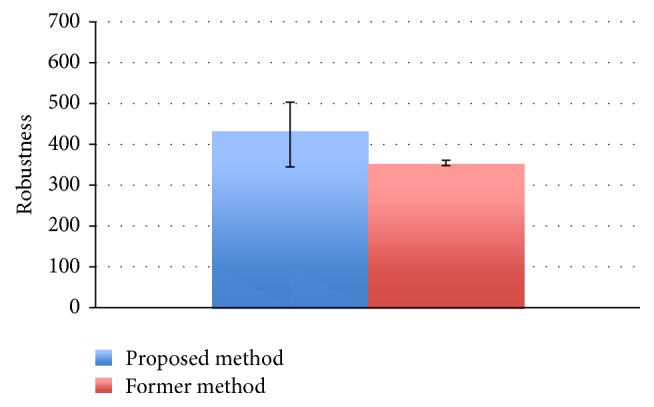
Comparison of robustness.

The results of the comparison experiments are shown in Figures 11 and 12.

Figure 11 shows the maximum and minimum values and the average value of the readability of the final solution by the former method and the proposed method. We calculated the result of the *t*-test with a significance level of 5% with respect to readability distribution, and it was found that there is significant difference in the distribution, with the proposed method improved in readability as compared to the former method.

**Figure 13:**
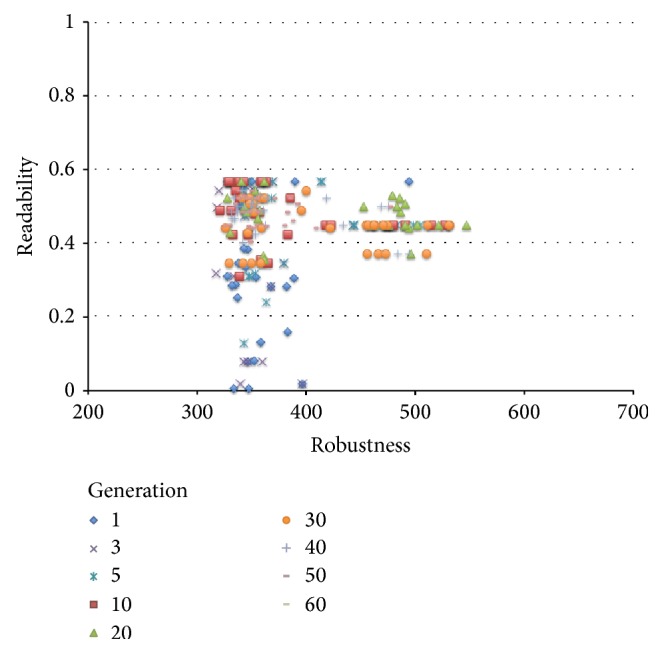
Sift of solutions using the proposed method.

**Figure 14:**
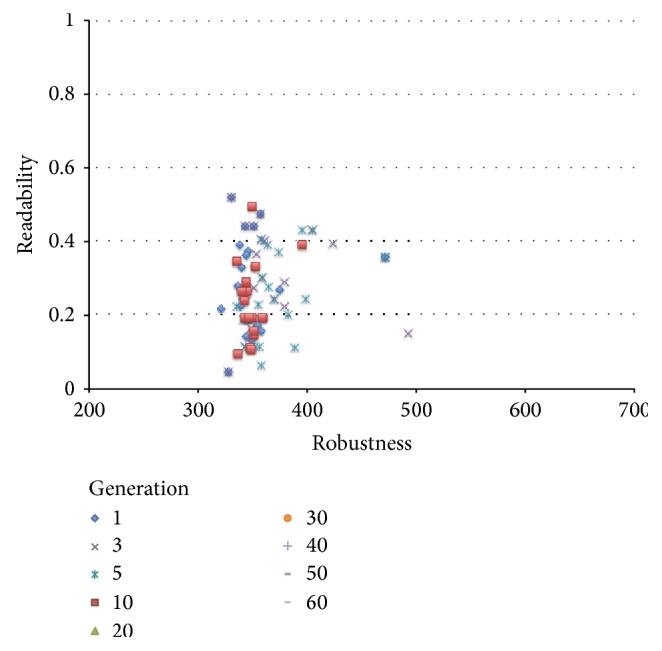
Sift of solutions using the former method.

Figure 12 shows the maximum and minimum values and the average value of the robustness of the final solution by the former method and the proposed method. As before, we calculated the result of the *t*-test with a significance level of 5% with respect to robustness distribution, and it was found that there is again an improvement with respect to robustness through the proposed method. Figures 13 and 14 plot the solution of each generation of the proposed and former method, respectively. From these figures, the proposed method has shown an improvement in both robustness and readability in parallel. In comparison, although a solution with high robustness given enough time does eventually come out through the former method, an improvement of readability does not. We have thus found that our method produces excellent results as compared to the former method.

## 5. Conclusion

Various systems in the real world can be modeled as multiagent systems, and appropriately designing the behavior rules of the agents is important to optimize the performance of that system. As important as improving robustness, however, is improving readability, for the sake of maintenance and operation. As a result, the object of the present study was the construction of a rule generation method that considers both robustness and readability.

In this paper, we proposed a combined method of PADO proposed by [18] and Operator Equalization proposed by [19] and used in [17], but incorporating metrics on readability from [16] on our the rule generation method. By applying our proposed method to the problem of aircraft ground control, it was possible to obtain a set of rules that was both more robust and more readable than the existing rule set, as well as when compared with the rules generated by the former method.

In the future, we hope to consider further discussion about other aspects of readability. In this paper, it was in the form of applying indicators of readability on the source code of generated rules, but a different point of view, such as ease of understanding, may become necessary when talking about humans successfully using a set of rules to act. Being able to calculate the usability of rules is necessary in order to further deepen such discussions.

## Figures and Tables

**Table 1:** Variables and these meanings.

Variable name	Meanings (source code)	Meanings (rule)
ProgramLength	Number of words in the documents	Number of nodes in trees

ProgramVocabulary	Number of different words in the documents	Number of variables and constant values

Lines	Number of lines	Number of nodes in the graph

*x*	A word	A variable or constant value
